# The impact of storage time on coagulation factor activity in FFP

**DOI:** 10.3389/fmed.2025.1676874

**Published:** 2025-10-01

**Authors:** Jie Gao, Juan Zhao, Luo Xu, Rui He, Mei Li, Li Wang, Xuemei Liang, Yan Shao, Rong Chen, ChunBo Xian, Ke Li, Rong He

**Affiliations:** ^1^Chongzhou People’s Hospital, Chengdu, Sichuan, China; ^2^College of Biomedical Engineering and National Engineering Research Center for Biomaterials, Sichuan University, Chengdu, Sichuan, China

**Keywords:** FFP, blood storage, coagulation factor activity, coagulation pathway, blood transfusion management

## Abstract

**Objective:**

This research aims to explore the change law of coagulation factor activity in fresh frozen plasma (FFP) with storage time under standard storage conditions, evaluate the quality change characteristics during its validity period, and provide scientific basis for optimizing the inventory management and turnover strategy of fresh frozen plasma in primary hospitals.

**Methods:**

FFP under standard storage conditions (≤−18 °C) was followed up for 1 year (from the date of collection), and the activated partial thromboplastin time (APTT), prothrombin time (PT), international normalized ratio (INR) and factor VIII (FVIII) activity at different time points were measured, and the results were analyzed statistically.

**Results:**

The PT and APTT of FFP showed an upward trend with the extension of storage time, and remained within the normal reference range, but the growth rate of PT increased after more than 300 days. The activity of FVIII decreased by 19.35% compared with the baseline level, and the qualified rate was only 72% after 120 days of storage.

**Conclusion:**

Under standard storage conditions, the coagulation function indexes (PT, APTT) of FFP remain normal, but the activity of some coagulation factors (such as FVIII) will decrease significantly with the extension of storage time and inventory management of fresh frozen plasma that been stored nearly 300 days should be enhanced.

## 1 Introduction

**Figure 1:**
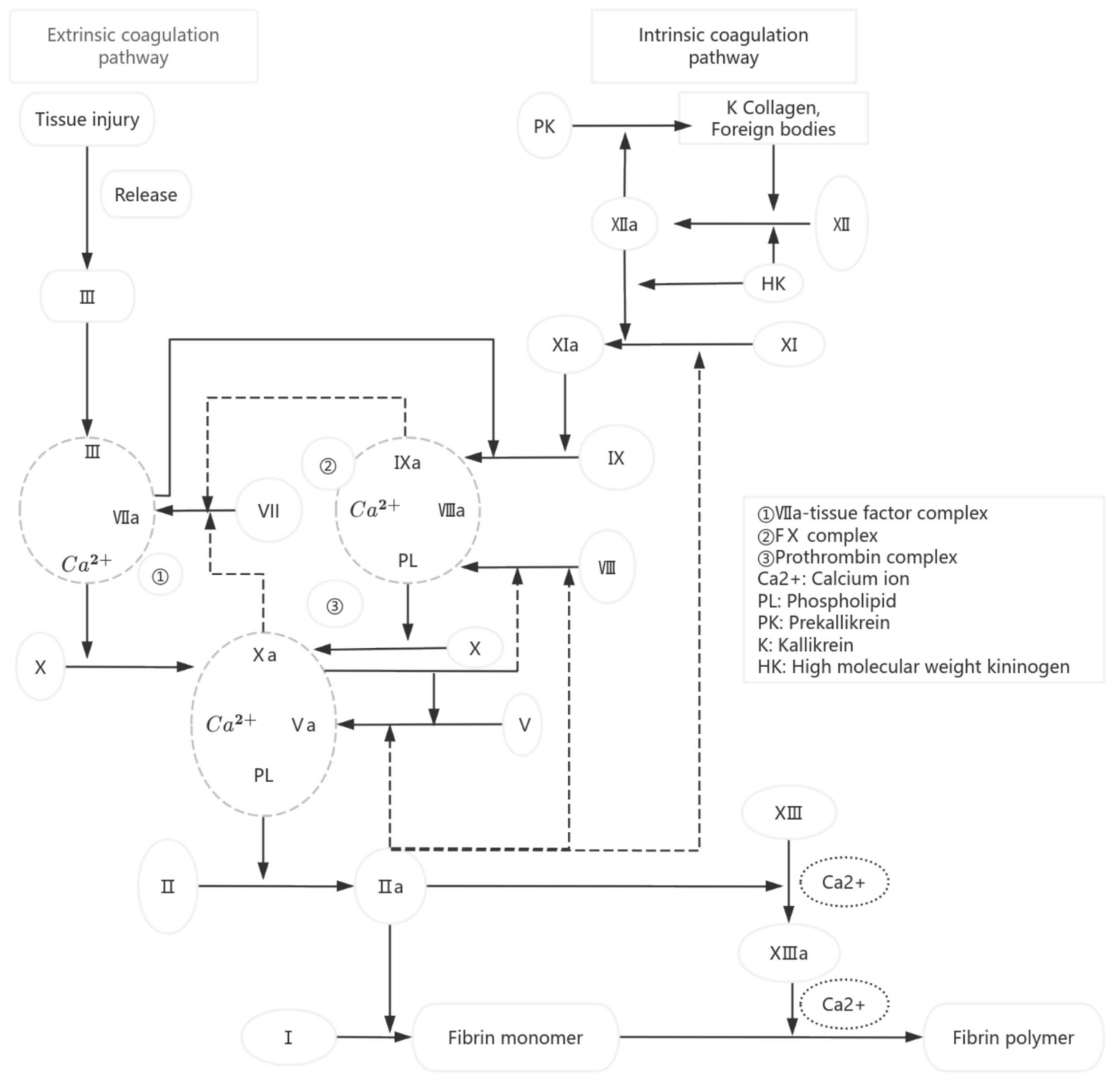
Diagram of coagulation pathway.

Blood coagulation is a cascade reaction process in which various coagulation factors are activated in turn, and finally fibrinogen is transformed into fibrin (Figure 1) (1, 2). As a key substance directly involved in the coagulation process, the lack or large loss of coagulation factors is the main cause of coagulation dysfunction in patients. Although blood transfusion is an important means of clinical treatment, it also has the risks of adverse reactions and spreading diseases, so six principles, such as irreplaceable, minimum dose, individualized infusion, safe infusion, reasonable infusion and effective infusion, must be strictly followed in clinical practice (3). As a common blood product in clinics, fresh frozen plasma (FFP) contains all coagulation factors, including unstable FV and FVIII, which is the main treatment to correct coagulation dysfunction in patients. FVIII is a key component in fresh frozen plasma, playing a crucial role in the blood coagulation process (4). The concentration of FVIII directly impacts the efficacy of clinical transfusions, making it a primary indicator for quality control (5, 6). It is worth noting that although most patients who receive FFP infusion do not have FVIII deficiency, FVIII is widely used as the key index of FFP quality control in the world at present: the European Commission requires that the average FVIII activity of FFP should be higher than 70% (7); The British standard stipulates that 75% of FFP samples should have a FVIII activity higher than 70% (8). “Quality requirements for whole blood and blood components” (GB18469-2012) also clearly stipulates that the FVIII activity in FFP shall not be less than 70%. This standard is mainly aimed at the quality control of blood station preparation. Previous studies have mostly focused on investigating the impact of preparation conditions on FFP quality or assessing the effects of post-thaw storage conditions and duration on FFP quality. However, there is still a lack of systematic research on the changes of FVIII activity and coagulation function indexes of FFP during the validity period under standard storage conditions. Based on this, this study systematically investigated the dynamic characteristics of coagulation factor activity in FFP by detecting the PT, APTT and FVIII activities of FFP at different storage times under standard storage conditions, and evaluated the influence of existing storage conditions on the quality of FFP, aiming at providing a scientific basis for the rational use of clinical FFP and the optimization of inventory management in primary hospitals. The research results are reported as follows:

## 2 Materials and methods

### 2.1 Sample source and test grouping

According to the Blood Station Technical Operating Procedures (Version 2019), the 118 whole blood samples were collected into CPDA-1 blood preservation solution (400 mL ± 40 mL whole blood corresponding to 56 mL preservation solution, 300 ± 30 mL whole blood corresponding to 42 mL preservation solution, 200 mL ± 20 mL whole blood corresponding to 28 mL preservation solution). All samples were strictly randomized to include all blood types, with no specified donor age or gender, collection site, or processing personnel. The 118 bags of FFP were prepared from the collected whole blood within 8 h, following the 2019 edition of the Blood Station Technical Operating Procedures. To control experimental variables, the study employed a dual-group control design: Group A (78 bags) had 7 portions of 4 mL samples each divided into disposable centrifuge tubes at the time of preparation. Group B (40 bags) had 7 portions of 15 mL samples each divided into dedicated plasma storage bags. After the samples were retained, the remaining plasma was re-quantified according to standard procedures by the Chengdu Blood Center and made available for normal clinical use. This grouping was designed to minimize waste of blood components, hence the maximum supply for Group B could only reach 40 units. The comparison group using disposable centrifuge tubes versus plasma storage bags excluded the potential impact of plasma sample retention methods on coagulation factor activity in this study. All samples were prepared and stored under the same conditions (≤−18 °C) and were randomly grouped for testing at predetermined time points (0, 60, 120, 180, 240, 300, and 360 days) to avoid interference with coagulation factor activity caused by repeated freeze–thaw cycles.

### 2.2 Instruments and reagents

The temperature monitoring was conducted using the Haier YB-HC00400 intelligent temperature acquisition system. Sample storage was performed in the Panasonic MDF-339 medical low-temperature freezer. The detection process utilized the Sysmex CA-7000 fully automatic coagulation analyzer and the KJX-IB type frozen plasma thawing box. All detection reagents and quality control materials were provided by Siemens Healthineers, and included the following: PT Detection Kit (Lot No.: 568112), APTT Detection Kit (Lot No.: 562712A), FVIII Detection Kit (Lot No.: 560845 A), PT quality control material (Lot No.: 507921), APTT quality control material (Lot No.:507921), and FVIII quality control material (Lot No.: 560842). All reagents were within their expiration dates, and calibration and internal quality control were performed before each test.

### 2.3 Method

#### 2.3.1 Plasma storage

In this study, upon arrival of the FFP samples at our laboratory, the pre-portioned specimens had been divided into seven batches. Each batch was arranged according to a fixed blood bag numbering system and placed into designated sample racks and plasma storage boxes. These were then stored in a Panasonic MDF-339 medical low-temperature freezer. The entire storage period strictly adhered to the requirements of the WS 399-2023 “Blood Storage Standard.” All 118 FFP samples were stored in a medical low-temperature refrigerator, and the temperature in the refrigerator was always kept within the specified range of ≤−18 °C. A designated person was responsible for monitoring and recording the freezer temperature daily and establishing a complete temperature monitoring archive.

#### 2.3.2 Detection method

FVIII activity was determined by means of the One-Stage Clotting Assay (OSA) using a standard curve with six dilution levels (3/2, 1/1, 1/2, 1/4, 1/8, 1/16) of reference standards (Siemens, Germany). The reference range was 70–150%. Both activated partial thromboplastin time (APTT) and prothrombin time (PT) were measured through coagulation methods. The reference range for APTT was 32–43 s, and that for PT was 11–13 s. For all reagents, intra-assay precision (coefficient of variation, CV ≤ 3.0%) and inter-assay precision (CV ≤ 10%) were ensured.

In this study, FFP samples were processed using the water bath method. To ensure the accuracy of the detection results, a grouped thawing strategy was employed, with 10 samples being processed simultaneously in each group. The specific procedure was as follows: The samples were placed in a water bath maintained at 37 °C, with strict control over the thawing time and real-time monitoring of the sample status. When the samples were completely thawed, with no visible ice crystals, no fibrin precipitation, and normal color, (Group A: 4 min ± 1 min; Group B: 5 min ± 1 min), the thawed FFP samples were transferred to the test cups, centrifuge immediately, and then tested for PT, APTT, and FVIII activity using the CA-7000 fully automatic coagulation analyzer. To minimize the impact of post-thaw storage time on the detection results, the operation of “thawing the next group only after completing the testing of the current group” was adopted. All tests were repeated three times, and the final results were obtained by averaging the three measurements.

#### 2.3.3 Detection scheme

**Figure 2:**
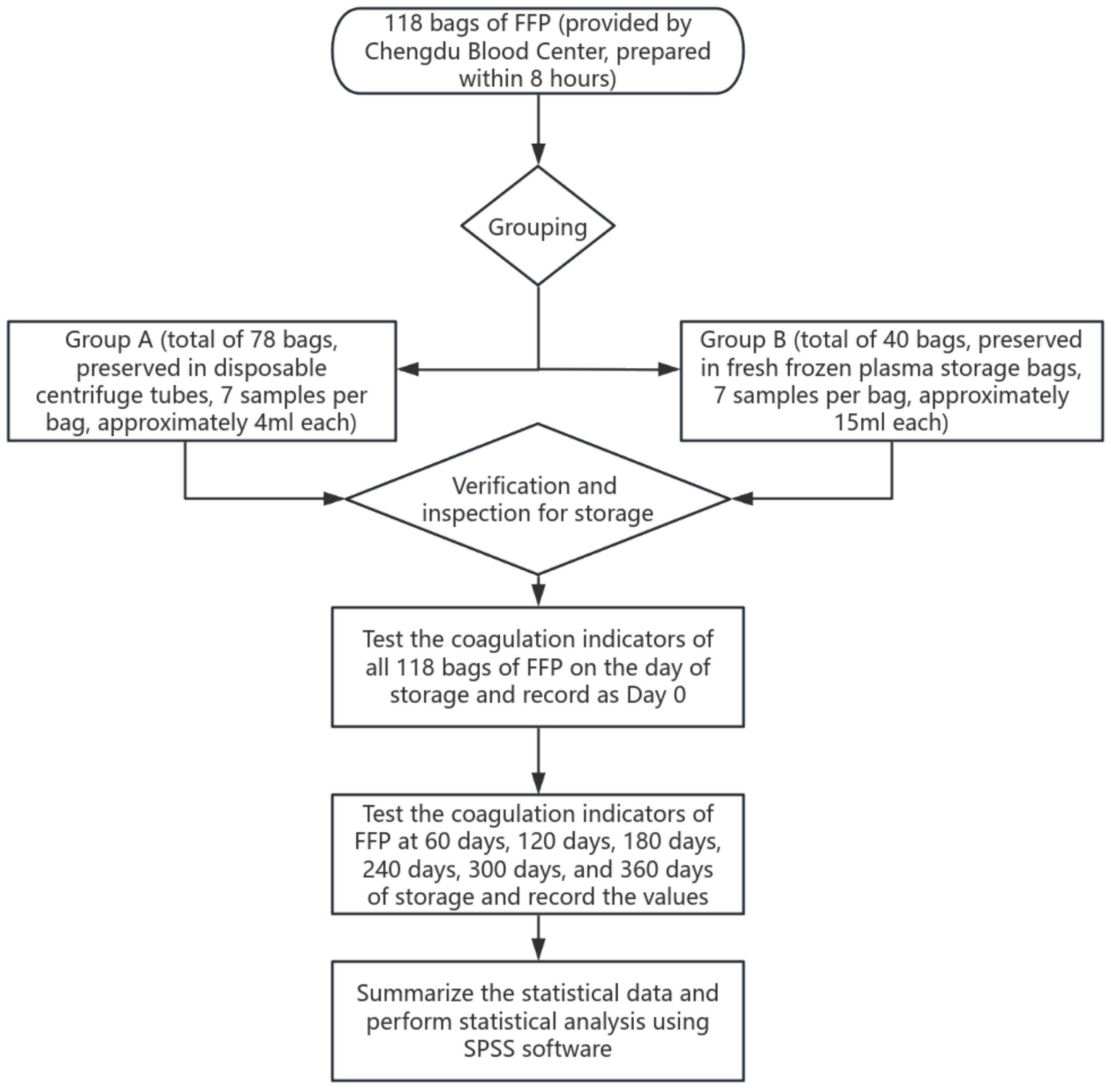
Flow chart of sample detection.

In this study, according to the WS 400-2023 “Blood Transport Standard,” the FFP samples passed the test in Chengdu Blood Center were divided into two groups (Group A and Group B) and transported to the laboratory. The transportation day was marked as the 0th day of storage, and the initial coagulation index was detected immediately. The obtained data were used as the baseline control values for subsequent storage period tests. Subsequently, systematic coagulation function tests were performed on the samples of both groups at predetermined time points, namely on Day 60, Day 120, Day 180, Day 240, Day 300, and Day 360 of storage, to dynamically observe the impact of different storage durations on plasma coagulation activity. All tests were completed under the same experimental conditions to ensure the comparability of the data (Figure 2).

### 2.4 Statistical processing

#### 2.4.1 Unit of measurement

The coagulation activity of FVIII is expressed in units of %, while PT and APTT are measured in seconds (s).

#### 2.4.2 Statistical analysis

All data were analyzed and processed using SPSS 25.0 statistical software. Quantitative data were presented in the form of mean ± standard deviation (X̅ ± SD). The comparison between the two sets of data (Group A and Group B) was expressed as the mean ± 2 standard errors of the mean (X̅ ± 2SEM), which approximates the 95% confidence interval. The changes in coagulation indices at different storage time points were assessed using multivariate repeated measures analysis of variance (ANOVA), and paired t-tests were employed to compare differences between experimental groups and differences among different test items at the same time point. Statistical significance was set at *p* < 0.05.

## 3 Results

### 3.1 Dynamic trends of coagulation FVIII: C (%), APTT(s), PT(s) and INR of FFP in group A and B during 360 days under standard storage conditions (≤−18 °C)

**Figure 3:**
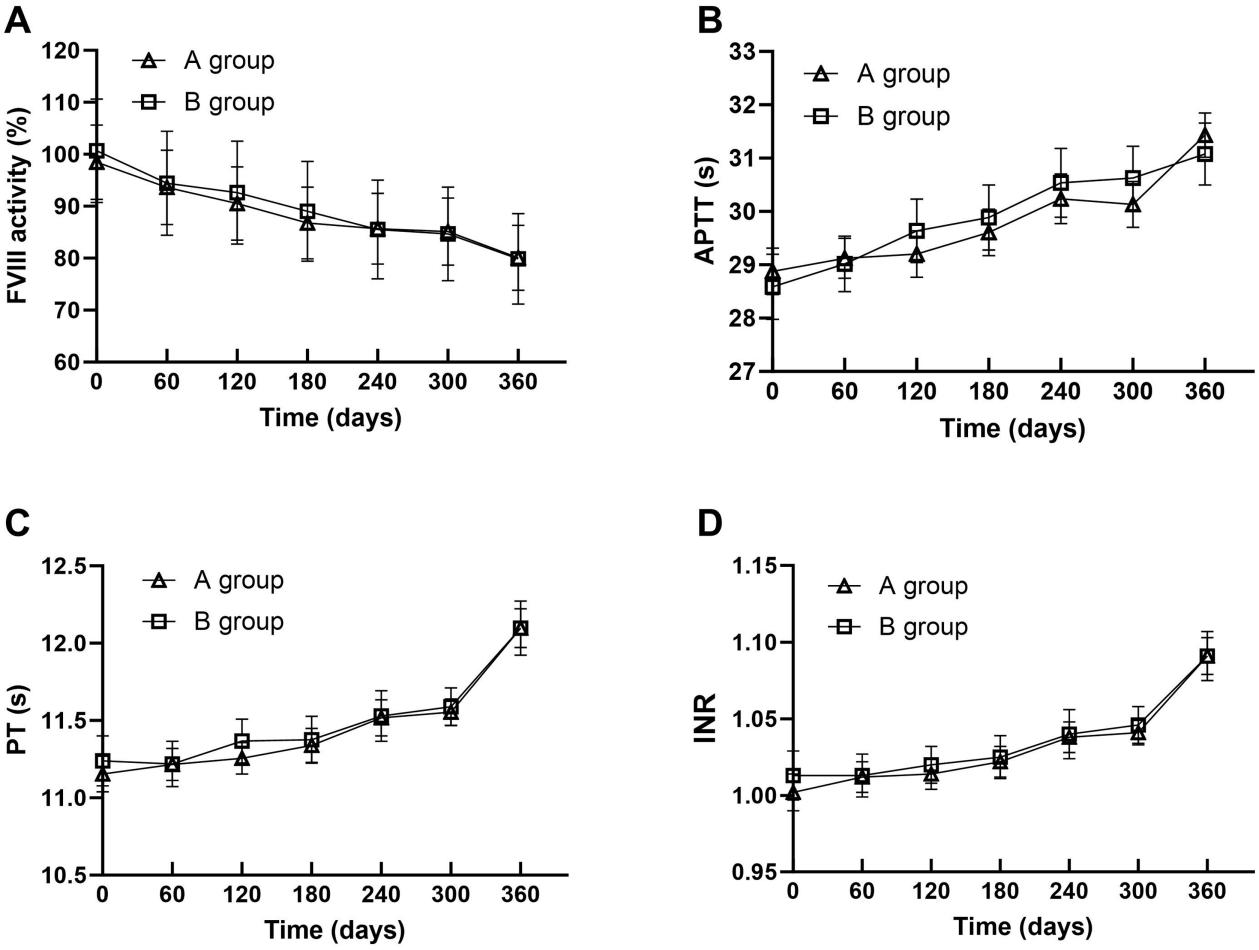
Trends in FVIII Activity **(A)**, APTT **(B)**, PT **(C)**, and INR **(D)** changes of FFP in Groups A and B under standard storage.

**Figure 4:**
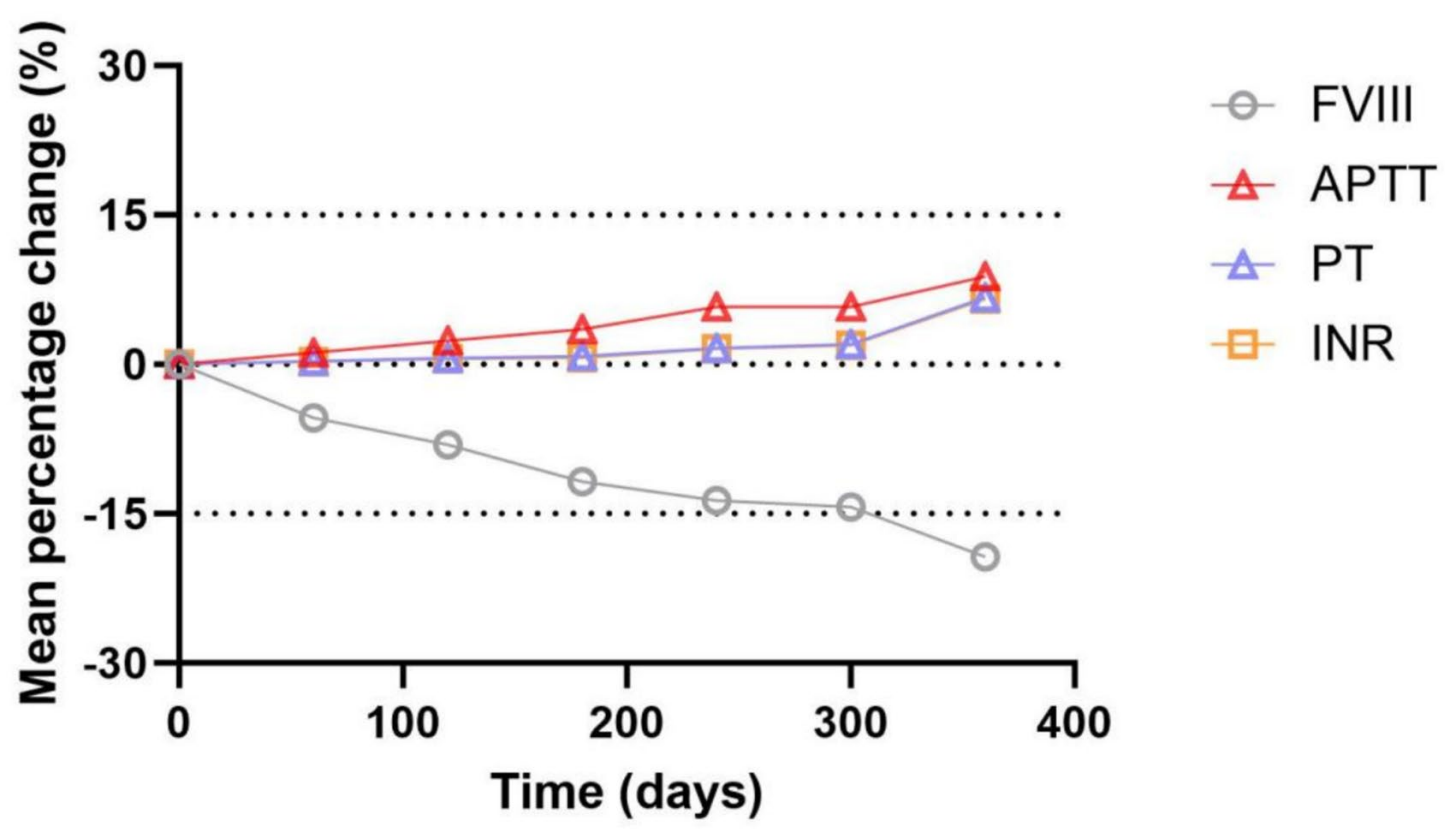
Trend plot for mean percentage changes in FVIII, APTT, PT and INR of FFP within 360 days under standard storage conditions.

Table 1 and Figures 3, 4 show the changes of FVIII activity (%), APTT(s), PT(s) and INR of FFP under standard storage conditions (≤−18 °C) for 360 days (Figure 3A). The FVIII activity of FFP in group A and group B showed a significant downward trend with the extension of storage time. Within 360 days, the FVIII activity in group A decreased by 18.7% and that in group B decreased by 20.8%, and there was no statistical difference between the two groups (*p* = 0.872) (Figure 3B). APTT(s) increased by 2.556 s in group A and 2.492 s in group B, and there was no statistical difference between the two groups (*p* = 0.735) (Figures 3C,D). Both PT (s) and INR showed a continuous upward trend with time. Before 300 days, the numerical changes at each time point were relatively gentle (the increase of PT was ≤0.18 s/60 days, and the increase of INR was ≤ 0.008/60 days), but after 300 days, the increase was obviously accelerated: the increase of PT in the later period was 1.3 times that of the previous period (0.543 s in group A, B). The increase of INR in the later period was 1.2 times that in the earlier period (0.050 in group A and 0.045 in group B, *p* = 0.450, with no statistical difference). Table 2 summarizes the mean values and 95% confidence intervals for FVIII: C (%), APTT(s), PT(s), and INR in 118 bags of FFP. The rate of change for each parameter was calculated relative to baseline values measured on storage Day 0. Comprehensive analysis showed that after 360 days of FFP storage, the activity of FVIII decreased by 19.34%, APTT prolonged by 8.82%, PT prolonged by 6.72%, and the growth rate of PT at 360 days was 1.3 times that at 300 days, and INR increased by 6.59% (Figure 4).

**Table 1:** Detection results of FVIII: C (%), APTT (s), PT (s), and INR in FFP of Groups A and B under standard storage conditions within 360 days (mean ± 2SEM).

Project		Time						
		0 days	60 days	120 days	180 days	240 days	300 days	360 days
FVIII: C	Group A	98.462 ± 7.142	93.616 ± 7.164	90.501 ± 7.076	86.759 ± 6.894	85.697 ± 6.822	85.145 ± 6.464	80.083 ± 6.244
	Group B	100.657 ± 9.974	94.426 ± 10.004	92.621 ± 9.882	89.015 ± 9.628	85.545 ± 9.526	84.685 ± 9.026	79.877 ± 8.718
APTT	Group A	28.879 ± 0.438	29.122 ± 0.374	29.205 ± 0.428	29.605 ± 0.438	30.238 ± 0.464	30.133 ± 0.428	31.435 ± 0.418
	Group B	28.588 ± 0.610	29.018 ± 0.522	29.638 ± 0.598	29.885 ± 0.612	30.535 ± 0.646	30.628 ± 0.598	31.080 ± 0.584
PT	Group A	11.154 ± 0.116	11.216 ± 0.104	11.255 ± 0.102	11.339 ± 0.110	11.518 ± 0.118	11.555 ± 0.086	12.098 ± 0.126
	Group B	11.239 ± 0.162	11.219 ± 0.146	11.367 ± 0.142	11.375 ± 0.152	11.530 ± 0.164	11.589 ± 0.122	12.098 ± 0.176
INR	Group A	1.002 ± 0.012	1.012 ± 0.010	1.014 ± 0.010	1.022 ± 0.010	1.038 ± 0.010	1.041 ± 0.008	1.091 ± 0.012
	Group B	1.013 ± 0.016	1.013 ± 0.014	1.020 ± 0.012	1.025 ± 0.014	1.040 ± 0.016	1.046 ± 0.012	1.091 ± 0.016

**Table 2:** Test results for FVIII: C (%), APTT (s), PT (s), and INR of 118 bags of FFP under standard storage conditions, along with their rate of change over storage time.

	FVIII			APTT			PT			INR		
Time	Average rate of change	Mean ± SD	2.5th–97.5th percentile	Average rate of change	Mean ± SD	2.5th–97.5th percentile	Average rate of change	Mean ± SD	2.5th–97.5th percentile	Average rate of change	Mean ± SD	2.5th–97.5th percentile
0 days	0%	99.206 ± 31.424	93.485–105.634	0%	28.781 ± 1.927	28.362–29.105	0.00%	11.183 ± 0.512	11.098–11.295	0%	1.006 ± 0.053	1.012–1.052
60 days	−5.36%	93.891 ± 31.505	87.928–100.114	1.18%	29.086 ± 1.646	28.752–29.388	0.31%	11.217 ± 0.461	11.128–11.307	0.29%	1.012 ± 0.043	1.019–1.051
120 days	−8.05%	91.220 ± 31.131	85.543–97.580	2.40%	29.352 ± 1.891	29.058–29.785	0.63%	11.293 ± 0.453	11.224–11.398	0.58%	1.016 ± 0.041	1.022–1.055
180 days	−11.78%	87.524 ± 30.336	82.023–93.751	3.55%	29.700 ± 1.933	29.372–30.118	0.82%	11.351 ± 0.482	11.264–11.450	0.68%	1.023 ± 0.044	1.024–1.055
240 days	−13.67%	85.646 ± 29.992	79.820–91.422	5.78%	30.339 ± 2.042	29.993–30.781	1.65%	11.522 ± 0.518	11.424–11.624	1.55%	1.038 ± 0.048	1.33–1.064
300 days	−14.33%	84.989 ± 28.422	79.418–90.412	5.76%	30.301 ± 1.898	30.016–30.745	2.03%	11.567 ± 0.382	11.498–11.646	1.94%	1.043 ± 0.035	1.041–1.064
360 days	−19.34%	80.014 ± 27.453	74.671–85.290	8.82%	31.314 ± 1.844	30.902–31.613	6.72%	12.098 ± 0.553	11.991–12.205	6.59%	1.091 ± 0.050	1.080–1.120

### 3.2 Under the standard storage condition (≤−18 °C), the number and proportion of plasma bags that meet the quality requirements (activity of FVIII > 70%) in FFP showed a dynamic change trend with storage time

In this research, the activity of FVIII > 70% was used as the quality standard of FFP (Table 3). It should be noted that although international clinical laboratories generally use international unit concentration (IU/100 mL or IU/mL) to report the activity of coagulation factors, this research uses the percentage of normal value to express the activity of FVIII. Because the standard plasma used in this research has passed the WHO standard calibration (1 IU is defined as the active amount in 1 mL fresh mixed normal plasma) (9), the activity percentage reported in this paper is equivalent to IU/100 mL unit. Under standard storage conditions, the number and percentage of qualified FFP bags decreased to below 75% after 60, 120, 180, 240, 300, and 360 days of storage, and the qualified rate further decreased to below 60% after 360 days of storage.

**Table 3:** Number and proportion of all tested fresh frozen plasma with FVIII content > 0.7 IU/mL over time under standard storage conditions.

Time	0 days	60 days	120 days	180 days	240 days	300 days	360 days
Total	118	118	118	118	118	118	118
Number of qualified	100	89	85	80	82	78	70
Qualified ratio	84.75%	75.42%	72.03%	67.80%	69.49%	66.10%	59.32%

## 4 Discussion

FFP, as an important clinical blood product, is mainly used to supplement bleeding or bleeding tendency caused by coagulation factor deficiency, and it is a key treatment for severe coagulation dysfunction and massive blood loss. In FFP quality control, the content of FVIII is generally used as the main evaluation index in the world (10). The guideline of European Commission stipulates that the average FVIII activity of FFP should be higher than 70%, and the British standard requires that 75% of plasma FVIII activity should be higher than 70%, while the national standard of our country also takes FVIII activity higher than 70% as the quality standard of FFP. It is worth noting that the activity of FVIII may be affected during preparation and storage because of its instability. The quality control requirement of FFP preparation process in China is that the preparation process is controlled if the FVIII activity of 75% in the sample is higher than 70% (11). The test results of FFP samples used in this study on the day of collection (day 0) showed that the qualified rate of FVIII activity reached 84.75% and the average activity was 99.206%, which met the quality standards. Regarding storage conditions, the American Association of Blood Banks (AABB) stipulates that FFP should be stored at a temperature of ≤−18 °C (12). Because except for a few unstable coagulation factors, most human coagulation factors can keep their activity stable at around −20 °C. However, many studies show that the optimal storage temperature of coagulation factors should be lower than −30°C (13), and its mechanism may be that when the temperature is higher than −23 °C, the mixed crystals of salts, protein and water formed by FFP at the initial stage of freezing will liquefy, thus affecting the activity of unstable factors (14). In this research, the change law of FVIII activity in FFP under standard storage conditions was systematically determined by strictly referring to the quality requirements of FFP in current guidelines. It is noteworthy that clinical studies have confirmed that coagulation factor activity within the range of 50 to 150% does not significantly affect coagulation function (15). Based on this discovery, this research not only detected the activity of FVIII, but also carried out the classic coagulation function tests such as APTT, PT and INR in order to evaluate the quality change of FFP under standard storage conditions more comprehensively and objectively. In order to ensure the reliability of the research results, this research adopts the grouping sample retention test: Group A samples are stored in disposable centrifuge tubes, which not only meets the ethical requirements of scientific research but also avoids the waste of blood resources; Group B samples were stored in standard FFP storage bags as control. The experimental data showed that there was no significant difference in the detection indexes between the two groups, which not only verified that the storage mode of centrifuge tube had no significant influence on the detection indexes of this study, but also provided a reliable methodological reference for subsequent related research.

Clinical coagulation detection is an important detection method to reflect the coagulation function of body by evaluating the rate of blood clot formation in coagulation cascade reaction. Among them, APTT, PT and INR, as the core detection indexes, are of key value in the diagnosis of secondary hemostasis dysfunction and the evaluation of coagulation factor deficiency. APTT detection mainly reflects the functional status of endogenous coagulation pathways (involving coagulation factors, XII, XI, IX and VIII) and common pathways (involving factors X, V, II and I). However, PT detection is highly sensitive to exogenous coagulation pathway (factor VII) and common pathway (factors II, V and X) and fibrinogen level. INR, as a standardized evaluation index of coagulation function, is calculated based on the international sensitivity index (ISI) of thromboplastin reagents and the measured PT value. ISI is an important parameter that reagent manufacturers pass strict calibration when they leave the factory, and the introduction of INR effectively eliminates the difference of PT value when different ISI reagents are used in different laboratories. This standardized treatment makes the INR results of the same specimen under different detection conditions highly comparable.

The data of this research showed that the qualified rate of FVIII activity (>70%) was significantly lower than 75% when FFP was stored for more than 120 days under the standard storage condition of ≤−18 °C. According to the quality requirements of the British Hematology Standards Committee (BCSH) guidelines, the quality of FFP was not up to the standard at this time. However, if the average FVIII activity is used as the evaluation index according to the European Commission guidelines, the average FVIII activity of FFP in this study only decreased by 8.05% after 120 days of storage, which is significantly lower than the reported value in the literature: Previous studies showed that the activity of FVIII decreased by more than 10% after 3 months’ storage at −24 °C (13), and the average change of FVIII activity reached 15% after 2 months’ storage at −15 °C to −25 °C (16). Due to the lack of research on coagulation factor and function testing during FFP storage in prior studies, the comparative data originated from frozen plasma samples freshly collected from healthy individuals and subsequently stored. As a result, variations in sample collection procedures, storage conditions, and plasma thawing protocols among studies probably constitute the primary factors contributing to the inconsistent decline rates of FVIII. In this study, the average activity of FVIII was maintained at 80% even after 360 days of storage, which was fully consistent with the requirements of the European Commission.

Previous research has shown that PT is mainly influenced by the activities of FV and FVIII, while APTT mainly reflects the functional states of FVIII and FXII (17). The experimental data showed that under the standard storage condition of ≤−18 °C, the activity of FVIII in FFP decreased gradually with the extension of storage time, corresponding to the continuous increase of APTT value, and there was a significant negative correlation between them. Considering that previous studies have confirmed the stability of FXII under low temperature storage conditions, the increase of APTT value can be considered to be caused by the loss of FVIII caused by long-term storage. Similarly, PT and INR also showed an upward trend with the storage time. Combined with the known stability characteristics of FVIII and the research results reported in the literature that the activity of FV decreased by about 16% after being stored at −15 °C to −25 °C for 1 month (17), it can be reasonably inferred that the prolongation of PT is mainly due to the decrease of the activity of FV. During the whole research cycle, APTT and PT increased by about 9% and 7% respectively, and the growth rate of PT reached 1.3 times in the late storage period (300–360 days), which suggested that 300 days might be the key time node for the accelerated decline of coagulation factor activity of FFP.

Based on the above findings, although FFP under standard storage conditions can still maintain the basic coagulation function within the validity period, its rate of quality degradation is obviously accelerated after more than 300 days. Therefore, it is suggested that FFP should be made available for clinical use within 300 days, adhering to the “first expired, first out (FEFO)” principle, to ensure the effect of blood transfusion treatment and optimize the utilization of blood resources.

The current study has the following limitations. First, the samples were obtained from voluntary blood donors at the Chengdu Blood Center, and we were unable to track their specific information. Consequently, we did not explore the impact of donor variability on initial coagulation factor levels; this study was a single-center investigation. Second, to avoid blood wastage, sample retention was limited. Only major coagulation functions and key factors were tested, with no investigation into other coagulation factors. Finally, most countries and regions lack clear clinical guidelines for evaluating the efficacy of plasma transfusion therapy. Consequently, it is challenging to directly correlate the findings of this study with clinical transfusion outcomes.

## Data Availability

The original contributions presented in the study are included in the article/supplementary material, further inquiries can be directed to the corresponding authors.
